# A Systematic Review of the Impact of Mitochondrial Variations on Male Infertility

**DOI:** 10.3390/genes13071182

**Published:** 2022-06-30

**Authors:** Houda Amor, Mohamad Eid Hammadeh

**Affiliations:** Department of Obstetrics & Gynecology, Biochemistry & Molecular Biology of Reproductive Medicine, University of Saarland, 66424 Homburg, Germany; mohamad.eid.hammadeh@uks.eu

**Keywords:** male infertility, mitochondria, mtDNA

## Abstract

According to current estimates, infertility affects one in four couples trying to conceive. Primary or secondary infertility can be due either to both partners or only to the man or the woman. Up to 15% of infertility cases in men can be attributed to genetic factors that can lead to irreversible partial or complete spermatogenic arrest. The increased use of assisted reproductive technology (ART) has provided not only insights into the causes of male infertility but also afforded a diagnostic tool to detect and manage this condition among couples. Genes control a variety of physiological attributes, such as the hypothalamic–pituitary–gonadal axis, development, and germ cell differentiation. In the era of ART, it is important to understand the genetic basis of infertility so as to provide the most tailored therapy and counseling to couples. Genetic factors involved in male infertility can be chromosome abnormalities or single-gene disorders, mitochondrial DNA (mtDNA) mutations, Y-chromosome deletions, multifactorial disorders, imprinting disorders, or endocrine disorders of genetic origin. In this review, we discuss the role of mitochondria and the mitochondrial genome as an indicator of sperm quality and fertility.

## 1. Mitochondrial DNA (mtDNA)

The mitochondrion was first defined by Von Kölliker in 1856, during his study of muscle tissue. About two billion years ago, mitochondria originated when a precursor to the modern eukaryotic cell engulfed an α-proteobacterium through an opportunistic relationship [1]. Since then, mitochondria have kept the double-membrane structure of their ancestors, but their overall shape and composition have changed consistently. Mitochondria have retained their genomes (mtDNA), reflecting their evolutionary origin as bacteria. While nuclear genes encode most mitochondrial proteins, some respiratory proteins and mitochondrial tRNAs are still encoded by the mitochondrial genome [2].

Another feature of mitochondria is the presence of specific ribosomes that allow localized protein synthesis [3].

mtDNA is present in stroma and is only transmitted through the female germline [4]. Sperm have only 100 mtDNA copies compared to eggs, which have 150,000 [5]. Mitochondria are located in the midsection of spermatozoa and have one copy of mtDNA per mitochondria [6,7,8].

Farge et al. (2019) reported that the gene structure and arrangement of mtDNA are highly conserved across different mammal species, including dolphins, bears, dogs, foxes, horses, llamas, mice, pandas, and many others. This includes approximately 16.6 kbs of close circular double-stranded DNA (heavy and light) strands [9].

Cristae, the main sites of mitochondrial energy conversion, are far into the matrix. ATP synthase in cristae membranes operates under a low pH gradient between the intermembrane space (pH 7.2–7.4) and the matrix (pH 7.9–8) [10].

There are four electron transport chain (ETC) complexes inside the inner membrane. Each ETC complex, except II, has genes encoded by the mitochondrial genome, while the rest of the ETC subunits are encoded by the nucleus. The mitochondrial respiratory chain consists of 13 mtDNA-encoded proteins. Complex I includes seven nicotinamide adenine dinucleotide hydride (*NADH*) dehydrogenase subunits: NADH dehydrogenase 1 (*ND1*), NADH dehydrogenase 2 (*ND2*), NADH dehydrogenase 3 (*ND3*), NADH dehydrogenase 4 (*ND4*), NADH dehydrogenase 4L (*ND4L*), NADH dehydrogenase 5 (*ND5*), and NADH dehydrogenase 6 (*ND6*); complex III contains cytochrome B (*CYB*); complex IV contains three subunits: cytochrome oxidase subunit I (*COX I*), cytochrome oxidase subunit II (*COX II*), and cytochrome oxidase subunit III (*COX III*), while complex V contains *ATPase 6* and *ATPase 8* [11].

Complex I is the main point of entry of electrons into the respiratory chain and is suggested as the rate-limiting step in general respiration. Therefore, it plays a critical role in energy metabolism [12]. For that reason, mitochondria change in location and number according to cell type. When they are present in large numbers in cells, this means that these cells require a lot of energy; for example, in oocytes, mitochondria can number up to 100,000 [10].

mtDNA is a naked molecule that lacks introns and histones, and both of its strands are transcribed to synthesize functional proteins. Due to asexual replication, very basic repair mechanisms, a lack of protective histones, and close proximity to free radical formation sites, mtDNA has a 10- to 20-fold higher mutation rate than nuclear DNA. Furthermore, mtDNA replicates rapidly in the absence of DNA repair machinery [7]. Thus, mtDNA is 100-fold more prone to mutation than nuclear DNA [13].

Scientists believe that human mtDNA is inherited only from the mother and that paternal mtDNA disappears after the cleavage stage [14].

In October 2018, Luo and his team provided evidence that paternal mtDNA can be passed on to offspring [15]. A recent study by Annis et al. (2019) dismissed this model of inheritance, which hinged on the notion of biparental mtDNA transmission to the offspring, as inaccurate [16].

## 2. Paternal Inheritance of Mitochondrial Genome (mtDNA)

Sperm-derived paternal mitochondria and their mtDNA reach the cytoplasm of an oocyte upon fertilization, typically disappear during early embryogenesis, and are never passed on to offspring.

However, the molecular mechanisms involved in this paternal mitochondrial clearance remain relatively unclear [17,18].

For decades, it has been agreed that human mtDNA is inherited entirely from the maternal line. The co-occurrence of mutant and wild-type variant alleles in the same individual (heterogeneity) and rapid changes in allele frequencies result in different disease severities in offspring [19].

Contrary to the long-held view that mtDNA is strictly inherited from the mother, a recent study challenges this view and provides evidence of additional paternal mtDNA transmission from father to offspring [15]. Paternal mtDNA is determined by quasi-Mendelian inheritance [16]. Furthermore, during intracytoplasmic sperm injection (ICSI), all sperm are injected into the cytoplasm of the oocyte and, interestingly, mtDNA is preserved, allowing offspring to share their father’s mtDNA [20].

A recent study provides evolutionary evidence in support of a role for paternal mtDNA in fertilization, with data collected from two neotropical primate families (Cebidae and Atelidae) suggesting that the midpiece-containing mtDNA has evolved to become larger and wider in the younger species (Atelidae) compared to the narrower and shorter midpiece in the ancestral species (Cebidae) [21].

A previous phenomenon supporting the role of sperm mtDNA in early embryonic development is the bi-uniparental inheritance of mitochondria in sea mussels, as females contain predominantly maternal mtDNA in their somatic cells, while males contain maternal mtDNA in their somatic cells and paternal mtDNA in gonads [22].

In a study performed by Cogswell et al. (2006), paternal mtDNA is seen to behave differently in eggs that will develop into males or females. In male-producing eggs, the paternal mtDNA tend to congregate together within the same cell, while in female-producing oocytes, the paternal mtDNA are scattered throughout the egg. During pre-cleavage stages of development, the paternal mitochondrion continues to have sex-specific roles and is seen to be localized in different ways, which supports its role in early development [23].

In a study on *Caenorhabditis elegans* (*C. elegans)*, researchers found that paternal mtDNA was completely destroyed after being fertilized by autophagosomes [24]. Another study showed that the entire sperm mtDNA was destroyed in the eggs of pigs and monkeys following fertilization by the ubiquitin–proteasome system, thanks to a specific microtubule-associated protein called SQSTM1 [25].

## 3. Mitochondrial Genome Mutations/Variations in Humans

Recently, many mtDNA mutations that are associated with the development and progression of human disease have been identified [26]. As many as 2 million Americans are affected by mitochondrial diseases (Lemonick, 2006). According to the United Mitochondrial Disease Foundation (UMDF), “every 30 min a child is born with mitochondrial disease which develops at age 10 [sic]”. Mitochondrial disease occurs in about 1 in 4000 people in the United States. The Center for Mitochondrial and Metabolic Diseases at UC San Diego estimates that 1000–4000 newborns in the United States are born with mitochondrial disease each year [27].

Numerous epidemiological studies supporting this idea have also been conducted in Europe [19,28,29,30].

Genetic variants in mitochondrial genes are associated with many diseases (Table 1). Most of these diseases affect organs with high energy demands, such as the brain, skeletal muscle, eyes, and heart [31]. Because mtDNA is not protected by histones or other DNA-binding proteins, it is more susceptible to DNA damage caused by excess reactive oxygen species (ROS) and free radicals in the matrix [32]. Furthermore, the mtDNA repair machinery is less efficient compared to that used in nuclear DNA repair [33]. Together, these elements increase the mutation rate in mtDNA by a factor of 10 to 100 compared to nuclear DNA [13].

A previous study found that the incidence of the 4216 T>C variant was higher in diabetic patients than in controls and was statistically associated with type 2 diabetes [34]. Another study found an interesting male-specific association between the 4216 T>C variant and infection rates, leading to complex sepsis and death [35].

Likewise, the 13708 G>A variant is associated with multiple clinical manifestations. It has been shown to increase susceptibility to multiple sclerosis [36], and, in another study, it was found to increase hereditary Leber‘s amplified expression in optic neuropathy (LHON) disease [37]. Males who carry the 13708 G>A variant have increased chances of developing Alzheimer’s disease. In fact, it was found that this variant is more dangerous in male patients than in female patients [38].

Recent studies have reported significant associations between *MTND3* polymorphisms and the risk of Parkinson’s disease, type 2 diabetes mellitus, and breast and esophageal cancer, but not gastric cancer [39,40,41]. Furthermore, among the identified *MTND3* single-nucleotide polymorphisms (SNPs), rs2853826 (A10398G) (*MT-ND3*) has been reported to be associated with the increased production of ROS in mitochondria, leading to oxidative stress and mtDNA damage [41].

Some mutations in the *MT-ND4L* gene have been found to be connected to specific disease disorders, such as LHON [42]. In the *MT-ND4* gene, variation is related to macular degeneration in the eye (AMD), mesial temporal lobe epilepsy (MTLE), cystic fibrosis, and even aging [43,44,45,46].

A significant association between rs28358280 (A10550G) (*MT-ND4L*) and body mass index (BMI) was identified for the first time with an increase in the G allele leading to higher BMI compared to when the allele alone was present [47]. Furthermore, rs2857285 is associated with aggressive ovarian cancer [48].

Four versions of the *ND6* gene were found to be correlated with Leigh disease in patients from Italy, France, and Germany: 14459G>A, 14495A>G, 14482C>A, and 14568C>T [42]. The variant 14439G>A was associated with a child who had mitochondrial respiratory chain disorder [49]. Another variant, 14459G>A, was found to be related to Leigh disease [50].

**Table 1:** Summary of studies reporting different mitochondrial disorders.

Mitochondrial Genome Abnormalities		Disease	References
Complex 1 NADH dehydrogenase	4216 T>C in *MT-ND 1* (missense variant)	Diabetes mellitus type 2 (T2D)	[51,52]
		Leber’s Hereditary Optic Neuropathy (LHON)	[53]
		Male-specific infection, leading to complicated sepsis and death	[35]
	5178 C>A in *MT-ND2*	T2D	[51]
	rs2853826 and rs414676521 in *MT-ND3*	Earlier age at onset in males, Machado–Joseph disease, breast cancer, T2D, Parkinson’s disease, esophageal cancer, gastric cancer, LHON	[39]
	120271 T>C and 12096 T>A in *MT-ND4*	Schizophrenia (SCZ), age-related muscular degeneration (AMD), mesial temporal lobe epilepsy (MTLE), cystic fibrosis	[43,44,45,46,54]
	rs28358280 in *MT-ND4L*	Body mass index	[47]
	rs2853495 in *MT-ND4L*	Ulcerative colitis and pancreatic cancer	[55,56]
	rs869096886 in *MT-ND4L*	SCZ	[57]
	rs2857285 in *MT-ND4L*	Ovarian cancer	[48]
	11777C>A in *MT-ND4L*	Late-onset encephalopathy	[58]
	13708 G>A in *MT-ND 5* gene	SCZ, increase in the susceptibility to multiple sclerosis, enhanced expression of LHON, increase in the risk of Alzheimer’s disease specifically in the male patients, breast cancer	[36,38,53,59,60,61,62]
	14439G>A in *MT-ND 6* gene (missense variant)	Mitochondrial respiratory chain disease	[49]
	14459 G>A in *MT-ND 6* gene	Leigh syndrome	[63]
	14459G>A, 14495A>G; 14482C>A and 14568C>T in *MT- ND 6*	LHON disease among patients from Germany, France, and Italy	[42]
Complex III *MT-CYB*	rs2853506 (15218A>G)	Epileptogenesis	[64]
	rs2853508	Breast cancer	[65,66]
	rs41518645	LHON	[67]
Complex IV	Mutations in *MT-CO III* genes	Recurrent myoglobinuria, LHON, severe encephalopathy, isolated myopathy	[68]
the rRNA: RNR 1 (12 S), RNR2 (16 S)	1709G>A, 15851A>G	Parkinson’s disease	[60,69]
tRNAs	Variant at position, 15928	Alzheimer’ disease	[70,71]
	8344 A>G in tRNA *Lys* gene	Myoclonus epilepsy and ragged–red fiber (MERRE) diseases	[72]
	8363 G>A in tRNA *Lys* gene	Correlated with autism spectrum disorders (ASD)	[73]
	8326 A>G in tRNA *Lys*, 15995 G>A in tRNA pro	Cystic fibrosis	[74]
ATP Synthetase 6 gene (*ATPase 6*)	9176 T>C	Mild myopathic change	[75]
	8839G>C	Retinitis pigmentosa syndrome (NARP)	[76]
	8914C>T	Mitochondrial encephalomyopathies	[77]
	8593 A>G	Leigh syndrome with a deficiency in mitochondrial energy production	[78]
ATP Synthetase 8 gene (*ATP 8*)	Mutations in *MT-ATP8*	LHON, MELAS, Leigh syndrome, NARP	[76,79]
*COII* gene	7750 C>A	SCZ	[80]

*MT-*: mitochondrial, *NADH*: nicotinamide adenine dinucleotide hydride dehydrogenase, *ND1*: NADH dehydrogenase 1, *ND2*: NADH dehydrogenase 2, *ND3*: NADH dehydrogenase 3, *ND4*: NADH dehydrogenase 4, *ND4L*: NADH dehydrogenase 4L, *ND5*: NADH dehydrogenase 5, and *ND6*: NADH dehydrogenase 6; *CYB:* Cytochrome b, *COX II*: cytochrome oxidase subunit II, and *COX III*: cytochrome oxidase subunit III.

## 4. Mitochondrial Genome Mutations/Variations and Male Infertility

It is estimated that approximately 15% to 30% of cases of male infertility are caused by genetic defects [81]. Mitochondria have their own genome (more than 93,000 genes). Some genes related to fertility and longevity are also known to be linked to mitochondria (Table 2).

Sperm have only 100 mtDNA copies compared to oocytes, which have 150,000 [82]. Spermatozoa rely on mitochondrial oxidative phosphorylation (OxPhos) machinery to generate the energy required for their motility [83].

Single-nucleotide polymorphisms or large deletions are types of mutations that affect sperm mtDNA. Thus, mutant mtDNA in sperm can lead to respiratory dysfunction that impairs energy production and results in decreased motility, which affects the normal activity of sperm [84,85,86,87].

Complex 1 plays a key role in OxPhos by receiving electrons from *NADH*. The captured energy of these electrons is used to release protons into the intermembrane space, which is subsequently used to generate ATP [88]. In addition, the mitochondrial genes *ATPase 6*, *ATPase 8*, *COX 3*, *COX 2,* *CYB, ND3*, *ND4*, *ND5*, and *ND6* play important roles in the construction of mature sperm and in progressive flagellar motility after ejaculation [87].

Therefore, pathogenic variants in the *ND* gene are expected to affect complex 1 activity, resulting in insufficient energy production, which will negatively affect sperm motility [84].

Large mtDNA deletions, including 4977 bp, 7345 bp, 7436 bp, and 7599 bp, have been associated with asthenozoospermia [89,90,91,92]. These deficiencies include deletions of several mitochondrial genes, namely *ATPase8* (lost at 7599 bp only), *ATPase 6*, *COX III*, *CYB*, and NADH dehydrogenase (*ND*) 3, *4*, *4L*, *5*, and *6*. These deletions also include the loss of eight tRNA genes [91]. Gene deficiencies from mtDNA deletions play critical roles in OxPhos in mitochondria; thus, their removal from mtDNA reduces energy conservation, which, in turn, negatively affects sperm-flagellar motility and contributes to asthenozoospermia [92].

The deletion of 4977 bp is considered the most common among mtDNA deficiencies [93]. This deletion involves the removal of seven genes and five transfer RNAs located between 8483 bp and 13459 bp in mtDNA, and the deleted site is located between two 13 bp repeats (5′-ACCTCCCTCACCA-3′) [94].

A previous study reported that sperm motility is inversely correlated with mtDNA deletion. According to the study’s results, mtDNA deletions were found in immobilized spermatozoa, whereas spermatozoa with normal motility did not have these deletions [95]. Moreover, it has been noted that the percentage of this deletion in mtDNA is higher in normal-motility sperm than in low-motility sperm [90].

Chari et al. (2015) found sperm with an abnormal motility which have a 4866 bp deletion in their mtDNA [96]. Another study found that 7436 bp sperm mtDNA deletions occurred more frequently in asthenozoospermic men than in normozoospermic men. Therefore, the use of this deletion as an indicator of reduced sperm motility has been proposed [89].

The change in mtDNA is likely to affect the speed and quality of sperm, according to studies performed by La Vignera et al. (2019). Their study showed that mtDNA alterations could have a significant impact on the quality of sperm as well as on its ability to swim [97].

In contrast, another study found no significant difference in mtDNA deletions in sperm from asthenozoospermic and normozoospermic men. Therefore, the researchers chose to ignore the role of these deletions in male infertility [14].

A previous study also showed that mtDNA deletions did not affect sperm motility, as the deletions did not differ significantly in the occurrence of mtDNA deficiencies in poor-quality and high-quality sperm [98].

A study in New Zealand comparing the incidence of mitochondrial variants in infertile and fertile men found 11 nucleotide substitutions in a group of infertile patients, compared with only 7 nucleotide substitutions in men with normal sperm. In addition, they identified two SNPs in *ATPase6*, and *ND4* mitochondrial genes were found to be associated with asthenozoospermia. Furthermore, 9055G>A occurred at a frequency of 10.7% in the ATP synthase 6 gene, and this missense variant resulted in the substitution of alanine by threonine [99].

In the Güney study, three different gene variants were identified in the *ND1* gene in patients who were infertile (C4159, G4153A, and T4114G). These gene variants were only found in patients who were unable to generate sperm, and not in patients who were able to produce sperm [100].

A previous study reported that the presence of massive mtDNA deletion is associated with asthenozoospermia because it results in the loss of some mitochondrial genes responsible for mitochondrial respiration, which provides sperm with the energy they need to move, thereby affecting male fertility [101].

An 11696G>A variant in the *MT-ND4* gene caused by a missense change was also found to be related to reduced sperm motility. This variant caused a change from valine at position 313 to isoleucine, which altered the structure of the protein [102].

Barbhuiya et al. (2016) found that the genes with the highest number of SNPs were the *ATPase 6* gene (21 SNPs), followed by the *ND2* gene (12 SNPs), and the *ATPase 8* gene (9 SNPs). The *ND4* gene showed the lowest number of SNP point mutations [103].

A genetic mutation causing a change in SNP T4216C in both fertile and infertile males was found by Khan et al. (2016) [104]. Zhang et al. also noted a decrease in the risk of asthenozoospermia for SNP C3398T, as it has a low frequency and small sample size [105]. Mughal et al. discovered that there is a strong link between the 15 bp deletion of *COX III* (at location 9390 to 9413) and infertility in men, with a P value of 0.033 [106].

Three different missense genetic variants were found in Complex I by Alsmadi et al. (2021) by screening mitochondrial genes *ND6*, *ND5*, *ND2*, and *ND1* in the sperm of male partners. The variants were 13708 G>A, 4216 T>C, and 12506T>A. These three variants were correlated negatively with sperm motility and ICSI outcomes. They were significantly different from wild types in terms of sperm motility, fertilization rate, embryo quality score, and median embryo cleavage score [107].

Recently, we scanned sterile and fertile males for polymorphisms by the direct sequencing of *MTND3*, *MTND4L*, and *MTND4* genes in our laboratory [108].

An SNP called rs2853495 in the *MTND4* gene was found to have an association with male infertility in the genotype frequency test. Moreover, G11719A (rs2853495) and A11251G (rs869096886) in gene *MTND4* were also tied to male infertility. Therefore, even if the genotype of the allele is different, the presence of the allele itself could still be tied to male infertility [109].

In addition, a total of 49 SNPs were identified and genotyped: 13 SNPs in *MT-CYB*, 14 SNPs in *MT-ATP6*, and 10 SNPs in *MT-ATP8* [110].

For three variants in *MT-CYB*, genotype frequencies were significantly different between fertile and sterile groups, and two SNPs showed a significant association between male infertility and allele frequencies: rs41504845 (C15833T) (*p* = 0.0147) and rs527236194 (T15784C) (*p* = 0.0014)). Furthermore, for *MT-CO3* and *MT-ATP6*, only rs7520428 showed statistically significant differences between the subfertile and fertile groups in both genotype and allele frequency tests (*p* < 0.0001 for both) [110].

**Table 2:** Summary of studies reporting different mitochondrial genome abnormalities associated with male infertility.

Mitochondrial Genome Abnormalities	Description	Effect on Male Infertility	References
4977 bp deletion	Most common deletion, located between 8483 bp and 13459 bp and characterized by the presence of two 13-bp repeated sequences (5′-ACCTCCCTCA CCA-3′)	Removal of seven genes and five tRNAs in mitochondrial DNA (mtDNA) associated with asthenozoospermia.	[90,93,111,112,113]
7599 bp deletion	Located between 8642 and 16243-bp and characterized by the presence of 7 nucleotides’ direct repeat (5′CATCAAC-3′) on both sides	-Removal of several mitochondrial genes: *ATP8* (lost with 7599 bp only), *ATP6*, cytochrome oxidase (*COX*) III, cytochrome b (*CYB*), NADH dehydrogenase (*ND*) *3,4, 4L, 5,* and *6.* -Reduction in the obtained energy, which in turn has a negative effect on sperm flagellum movement and leads to asthenozoospermia.	[91,92]
7345 bp deletion	Located between 9009 and 1654-bp		
4216 T>G	Located on *MT-ND1* gene	Negative correlation with sperm motility.	[107]
3243A>G		Positive correlation with the mt DNA copy number in embryo after ICSI as an adaptation for inefficient ATP production via oxidative phosphorylation due to mutated mtDNA.	[114]
A point mutation in the *ND1* gene at locus 4216		Association with recurrent pregnancy loss.	[115]
Copy-number variations (CNV)		mtDNA copy number affects implantation rate after ICSI.	[116]
11719G>A	Located on *MT-ND4* gene	Association with male infertility.	[109]
11251A>G			
9055 G>A		Association with poor sperm quality.	[99]
11696G>A (missense variant)		Association with reduced sperm motility.	[102]
11719 G>A		Association with poor semen quality.	[99]
11994 C>T (missense variant)		Negative correlation with sperm motility.	[117]
12506T>A	Located on *MT-ND5* gene	Negative correlation with sperm motility.	[107]
13708 G>A (missense variant)			
14172 T>C	Located on *MT-ND6* gene	Significant difference between the total fertilization failure group and control.	[118]
14368 C>T			
G15301A	Located on *MT-CYB*	These SNPs showed a statistically significant link to male infertility.	[110]
A 15326G			
A 15487 T			
15 bp deletion of cytochrome c oxidase III	Location 9390 to 9413	This deletion linked to human male infertility.	[106]

*MT-*: mitochondrial, *NADH*: nicotinamide adenine dinucleotide hydride dehydrogenase, *ND1*: NADH dehydrogenase 1, *ND3*: NADH dehydrogenase 3, *ND4*: NADH dehydrogenase 4, *ND4L*: NADH dehydrogenase 4L, *ND5*: NADH dehydrogenase 5, and *ND6*: NADH dehydrogenase 6; *CYB:* Cytochrome b, and *COX III*: cytochrome oxidase subunit III.

## 5. Mitochondrial Genome Mutations/Variations and Fertilization/Pregnancy Outcomes

Until a few years ago, maternally restricted mtDNA inheritance was the only accepted idea because paternal mtDNA disappears after the embryonic cleavage stage [14]. In 2018, Luo et al. presented strong proof of biparental mtDNA inheritance, showing evidence of parental mtDNA transmission from father to offspring according to mitochondrial disease inheritance patterns in three independent multigenerational families [15]. Ecker found that sons with ICSI had the same SNPs in mitochondrial genes (COX1, ND1, ND4, and ND5) as their fathers [20]. Paternal mtDNA similarity is sometimes as high as 99% [20].

Another study observed that mtDNA myopathy could be passed from father to son by ICSI and found that sperm mtDNA mutations were retained in the embryo [119]. This suggests that, at least in some cases, paternal mtDNA can be transmitted to offspring. Paternal mtDNA mutations can be diluted due to mitochondrial heterogeneity, as maternal mtDNA copies are much more numerous than paternal mtDNA [120].

Diez-Juan et al. demonstrated a strong correlation between implantation rate and mtDNA copy number in euploid embryos. They found that successfully implanted embryos had lower mtDNA content; this result applies to embryo transfer at the division or blastocyst stage. On the other hand, poorly implanted embryos are estimated to have high mtDNA copy numbers. Therefore, researchers believe that mtDNA copy number can be reliably used to predict successful engraftment [116].

A study of a Chinese population found that men with haplogroup Z were more prone to IVF failure, but fertilization was observed between fertile and infertile cohorts with haplogroup D or haplogroup G. There was no significant difference in failure. Therefore, it was concluded that the SNPs of the *MT-ND2* gene were not associated with overall fertility [121].

In a recent study, variations in mtDNA correlated with lower levels of embryo development compared to quality at the blastocyst stage. The quality of the embryo at the blastocyst stage correlated with better sperm motility [122].

Forty-five *ND6* gene variants were found, two of which had a significant difference in total fertilization failure between the groups studied [118].

Alsmadi et al. (2021) found that pregnancy rates were negatively affected by the presence of 4216T>C in *ND1*, and 13708G>A and 12506T>A variants in *ND5*. The lowest pregnancy rates were due to men with severe asthenozoospermia and mtDNA SNPs, while the highest pregnancy rates were due to normozoospermic men without mtDNA SNPs [107].

These results are consistent with a previous study that found a point mutation in the T>C variant at locus 4216 in the *ND1* gene to be associated with recurrent miscarriage [115].

One variant in the *ND4* gene was investigated in two separate studies. In one of them, a missense variant, 11994C>T, was found to be strongly associated with oligoasthenozoospermia in India [117]. In the other study, no association was found between this variant and oligoasthenozoospermia [123].

## 6. Conclusions

Human male infertility is a complex phenotype. Research into the field of genetics associated with this phenotype is increasing, and many genes, including their mutations and/or deletions, have been identified. In addition, mitochondria and their genomes have received attention due to their importance in sperm function and successful fertilization. To date, various mitochondrial variants associated with male infertility have been observed. However, more research is needed to understand how these changes lead to adverse outcomes in men.

## References

[1] Martin W.F., Müller M. (2007). Origin of Mitochondria and Hydrogenosomes.

[2] Andersson S.G.E., Zomorodipour A., Andersson J.O., Sicheritz-Pontén T., Alsmark U.C.M., Podowski R.M., Näslund A.K., Eriksson A.-S., Winkler H.H., Kurland C.G. (1998). The Genome Sequence of Rickettsia Prowazekii and the Origin of Mitochondria. Nature.

[3] St. John J.C., Sakkas D., Barratt C.L.R. (2000). A Role for Mitochondrial DNA and Sperm Survival. J. Androl..

[4] Chiaratti M.R., Macabelli C.H., Neto J.D.A., Grejo M.P., Pandey A.K., Perecin F., Collado M. (2020). Del Maternal Transmission of Mitochondrial Diseases. Genet. Mol. Biol..

[5] Sasarman F., Brunel-Guitton C., Antonicka H., Wai T., Shoubridge E.A., Allen B., Burelle Y., Charron G., Coderre L., DesRosiers C. (2010). LRPPRC and SLIRP Interact in a Ribonucleoprotein Complex That Regulates Posttranscriptional Gene Expression in Mitochondria. Mol. Biol. Cell.

[6] Kumar R., Venkatesh S., Kumar M., Tanwar M., Shasmsi B., Gupta P., Sharma R.K., Talwar P., Dada R. (2009). Oxidative Stress and Sperm Mitochondrial DNA Mutation in Idiopathic Oligoasthenozoospermic Men. Indian J. Biochem. Biophys..

[7] Shamsi M.B., Kumar R., Bhatt A., Bamezai R.N.K., Kumar R., Gupta N.P., Das T.K., Dada R. (2008). Mitochondrial DNA Mutations in Etiopathogenesis of Male Infertility. Indian J. Urol..

[8] Venkatesh S., Deecaraman M., Kumar R., Shamsi M.B., Dada R. (2009). Role of Reactive Oxygen Species in the Pathogenesis of Mitochondrial DNA (MtDNA) Mutations in Male Infertility. Indian J. Med. Res..

[9] Farge G.É.R., Falkenberg M. (2019). Organization of DNA in Mammalian Mitochondria. Int. J. Mol. Sci..

[10] Scheffler I.E. (2007). Mitochondria.

[11] Smeitink J., Van Den Heuvel L., DiMauro S. (2001). The Genetics and Pathology of Oxidative Phosphorylation. Nat. Rev. Genet..

[12] Sharma L., Lu J., Bai Y. (2009). Mitochondrial Respiratory Complex I: Structure, Function and Implication in Human Diseases. Curr. Med. Chem..

[13] O’Connell M., McClure N., Lewis S.E.M. (2002). A Comparison of Mitochondrial and Nuclear DNA Status in Testicular Sperm from Fertile Men and Those with Obstructive Azoospermia. Hum. Reprod..

[14] Cummins J.M., Jequier A.M., Martin R., Mehmet D., Goldblatt J. (1998). Semen Levels of Mitochondrial DNA Deletions in Men Attending an Infertility Clinic Do Not Correlate with Phenotype. Int. J. Androl..

[15] Luo S., Valencia C.A., Zhang J., Lee N.C., Slone J., Gui B., Wang X., Li Z., Dell S., Brown J. (2018). Biparental Inheritance of Mitochondrial DNA in Humans. Proc. Natl. Acad. Sci. USA.

[16] Annis S., Fleischmann Z., Khrapko M., Franco M., Wasko K., Woods D., Kunz W.S., Ellis P., Khrapko K. (2019). Quasi-Mendelian Paternal Inheritance of Mitochondrial DNA: A Notorious Artifact, or Anticipated Behavior?. Proc. Natl. Acad. Sci. USA.

[17] Sato M., Sato K. (2012). Maternal Inheritance of Mitochondrial DNA. Autophagy.

[18] Sato M., Sato K. (2013). Maternal Inheritance of Mitochondrial DNA by Diverse Mechanisms to Eliminate Paternal Mitochondrial DNA. Biochim. Biophys. Acta Mol. Cell Res..

[19] Wei W., Chinnery P.F. (2020). Inheritance of Mitochondrial DNA in Humans: Implications for Rare and Common Diseases. J. Intern. Med..

[20] Eker C., Celik H.G., Balci B.K., Gunel T. (2019). Investigation of Human Paternal Mitochondrial DNA Transmission in ART Babies Whose Fathers with Male Infertility. Eur. J. Obstet. Gynecol. Reprod. Biol..

[21] Steinberg E.R., Sestelo A.J., Ceballos M.B., Wagner V., Palermo A.M., Mudry M.D. (2019). Sperm Morphology in Neotropical Primates. Animals.

[22] Cao L., Kenchington E., Zouros E., Rodakis G.C. (2004). Evidence That the Large Noncoding Sequence Is the Main Control Region of Maternally and Paternally Transmitted Mitochondrial Genomes of the Marine Mussel (*Mytilus* Spp.). Genetics.

[23] Cogswell A.T., Kenchington E.L.R., Zouros E. (2011). Segregation of Sperm Mitochondria in Two- and Four-Cell Embryos of the Blue Mussel Mytilus Edulis: Implications for the Mechanism of Doubly Uniparental Inheritance of Mitochondrial DNA. Genome.

[24] Sato M., Sato K. (2011). Degradation of Paternal Mitochondria by Fertilization-Triggered Autophagy in *C. elegans* Embryos. Science.

[25] Song W.H., Yi Y.J., Sutovsky M., Meyers S., Sutovsky P. (2016). Autophagy and Ubiquitin-Proteasome System Contribute to Sperm Mitophagy after Mammalian Fertilization. Proc. Natl. Acad. Sci. USA.

[26] Ryzhkova A.I., Sazonova M.A., Sinyov V.V., Galitsyna E.V., Chicheva M.M., Melnichenko A.A., Grechko A.V., Postnov A.Y., Orekhov A.N., Shkurat T.P. (2018). Mitochondrial Diseases Caused by MtDNA Mutations: A Mini-Review. Ther. Clin. Risk Manag..

[27] Ahuja A.S. (2018). Understanding Mitochondrial Myopathies: A Review. PeerJ.

[28] Skladal D., Bernier F.P., Halliday J.L., Thorburn D.R. (2000). Birth Prevalence of Mitochondrial Respiratory Chain Defects in Children. J. Inherit. Metab. Dis..

[29] Darin N., Oldfors A., Moslemi A.R., Holme E., Tulinius M. (2001). The Incidence of Mitochondrial Encephalomyopathies in Childhood: Clinical Features and Morphological, Biochemical, and DNA Abnormalities. Ann. Neurol..

[30] Gorman G.S., Chinnery P.F., DiMauro S., Hirano M., Koga Y., McFarland R., Suomalainen A., Thorburn D.R., Zeviani M., Turnbull D.M. (2016). Mitochondrial Diseases. Nat. Rev. Dis. Prim..

[31] Frazier A.E., Thorburn D.R., Compton A.G. (2019). Mitochondrial Energy Generation Disorders: Genes, Mechanisms, and Clues to Pathology. J. Biol. Chem..

[32] Piomboni P., Focarelli R., Stendardi A., Ferramosca A., Zara V. (2012). The Role of Mitochondria in Energy Production for Human Sperm Motility. Int. J. Androl..

[33] Stewart J.B., Chinnery P.F. (2015). The Dynamics of Mitochondrial DNA Heteroplasmy: Implications for Human Health and Disease. Nat. Rev. Genet..

[34] Crispim D., Canani L.H., Gross J.L., Tschiedel B., Souto K.E.P., Roisenberg I. (2006). The European-Specific Mitochondrial Cluster J/T Could Confer an Increased Risk of Insulin-Resistance and Type 2 Diabetes: An Analysis of the m.4216T > C and m.4917A > G Variants. Ann. Hum. Genet..

[35] Gomez R., O’Keeffe T., Chang L.Y., Huebinger R.M., Minei J.P., Barber R.C. (2009). Association of Mitochondrial Allele 4216C with Increased Risk for Complicated Sepsis and Death after Traumatic Injury. J. Trauma Inj. Infect. Crit. Care.

[36] Yu X., Koczan D., Sulonen A.M., Akkad D.A., Kroner A., Comabella M., Costa G., Corongiu D., Goertsches R., Camina-Tato M. (2008). MtDNA Nt13708A Variant Increases the Risk of Multiple Sclerosis. PLoS ONE.

[37] Brown M.D., Starikovskaya E., Derbeneva O., Hosseini S., Allen J.C., Mikhailovskaya I.E., Sukernik R.I., Wallace D.C. (2002). The Role of MtDNA Background in Disease Expression: A New Primary LHON Mutation Associated with Western Eurasian Haplogroup. J. Hum. Genet..

[38] Maruszak A., Canter J.A., Styczyńska M., Zekanowski C., Barcikowska M. (2009). Mitochondrial Haplogroup H and Alzheimer’s Disease—Is There a Connection?. Neurobiol. Aging.

[39] Jin E.H., Sung J.K., Lee S.I., Hong J.H. (2018). Mitochondrial NADH Dehydrogenase Subunit 3 (MTND3) Polymorphisms Are Associated with Gastric Cancer Susceptibility. Int. J. Med. Sci..

[40] Van Der Walt J.M., Nicodemus K.K., Martin E.R., Scott W.K., Nance M.A., Watts R.L., Hubble J.P., Haines J.L., Koller W.C., Lyons K. (2003). Mitochondrial Polymorphisms Significantly Reduce the Risk of Parkinson Disease. Am. J. Hum. Genet..

[41] Pezzotti A., Kraft P., Hankinson S.E., Hunter D.J., Buring J., Cox D.G. (2009). The Mitochondrial A10398G Polymorphism, Interaction with Alcohol Consumption, and Breast Cancer Risk. PLoS ONE.

[42] Achilli A., Iommarini L., Olivieri A., Pala M., Hooshiar Kashani B., Reynier P., La Morgia C., Valentino M.L., Liguori R., Pizza F. (2012). Rare Primary Mitochondrial DNA Mutations and Probable Synergistic Variants in Leber’s Hereditary Optic Neuropathy. PLoS ONE.

[43] Valdivieso Á.G., Marcucci F., Taminelli G., Guerrico A.G., Álvarez S., Teiber M.L., Dankert M.A., Santa-Coloma T.A. (2007). The Expression of the Mitochondrial Gene MT-ND4 Is Downregulated in Cystic Fibrosis. Biochem. Biophys. Res. Commun..

[44] Gurses C., Azakli H., Alptekin A., Cakiris A., Abaci N., Arikan M., Kursun O., Gokyigit A., Ustek D. (2014). Mitochondrial DNA Profiling via Genomic Analysis in Mesial Temporal Lobe Epilepsy Patients with Hippocampal Sclerosis. Gene.

[45] Wallace D.C., Singh G., Lott M.T., Hodge J.A., Schurr T.G., Lezza A.M.S., Elsas L.J., Nikoskelainen E.K. (1988). Mitochondrial DNA Mutation Associated with Leber’s Hereditary Optic Neuropathy. Science.

[46] Restrepo N.A., Mitchell S.L., Goodloe R.J., Murdock D.G., Haines J.L., Crawford D.C. (2015). Mitochondrial Variation and the Risk of Age-Related Macular Degeneration across Diverse Populations. Pacific Symposium on Biocomputing Co-Chairs.

[47] Flaquer A., Baumbach C., Kriebel J., Meitinger T., Peters A., Waldenberger M., Grallert H., Strauch K. (2014). Mitochondrial Genetic Variants Identified to Be Associated with BMI in Adults. PLoS ONE.

[48] Earp M.A., Brooks-Wilson A., Cook L., Le N. (2013). Inherited Common Variants in Mitochondrial DNA and Invasive Serous Epithelial Ovarian Cancer Risk. BMC Res. Notes.

[49] Uehara N., Mori M., Tokuzawa Y., Mizuno Y., Tamaru S., Kohda M., Moriyama Y., Nakachi Y., Matoba N., Sakai T. (2014). New MT-ND6 and NDUFA1 Mutations in Mitochondrial Respiratory Chain Disorders. Ann. Clin. Transl. Neurol..

[50] Ronchi D., Cosi A., Tonduti D., Orcesi S., Bordoni A., Fortunato F., Rizzuti M., Sciacco M., Collotta M., Cagdas S. (2011). Clinical and Molecular Features of an Infant Patient Affected by Leigh Disease Associated to m.14459G > A Mitochondrial DNA Mutation: A Case Report. BMC Neurol..

[51] Jiang Z., Teng L., Zhang S., Ding Y. (2020). Mitochondrial ND1 T4216C and ND2 C5178A Mutations Are Associated with Maternally Transmitted Diabetes Mellitus. Mitochondrial DNA A.

[52] Crispín-Trebejo B., Robles-Cuadros M.C., Bernabé-Ortiz A. (2015). Association between Depression and Glycemic Control among Type 2 Diabetes Patients in L Ima, P Eru. Asia-Pac. Psychiatry.

[53] Lodi R., Montagna P., Cortelli P., Iotti S., Cevoli S., Carelli V., Barbiroli B. (2000). ‘Secondary’ 4216/ND1 and 13708/ND5 Leber’s Hereditary Optic Neuropathy Mitochondrial DNA Mutations Do Not Further Impair in Vivo Mitochondrial Oxidative Metabolism When Associated with the 11778/ND4 Mitochondrial DNA Mutation. Brain.

[54] Martorell L., Segués T., Folch G., Valero J., Joven J., Labad A., Vilella E. (2006). New Variants in the Mitochondrial Genomes of Schizophrenic Patients. Eur. J. Hum. Genet..

[55] Wang L., Bamlet W.R., De Andrade M., Boardman L.A., Cunningham J.M., Thibodeau S.N., Petersen G.M. (2007). Mitochondrial Genetic Polymorphisms and Pancreatic Cancer Risk. Cancer Epidemiol. Biomark. Prev..

[56] Dankowski T., Schröder T., Möller S., Yu X., Ellinghaus D., Bär F., Fellermann K., Lehnert H., Schreiber S., Franke A. (2016). Male-Specific Association between MT-ND4 11719 A/G Polymorphism and Ulcerative Colitis: A Mitochondria-Wide Genetic Association Study. BMC Gastroenterol..

[57] Gonçalves V.F., Giamberardino S.N., Crowley J.J., Vawter M.P., Saxena R., Bulik C.M., Yilmaz Z., Hultman C.M., Sklar P., Kennedy J.L. (2018). Examining the Role of Common and Rare Mitochondrial Variants in Schizophrenia. PLoS ONE.

[58] Deschauer M., Bamberg C., Claus D., Zierz S., Turnbull D.M., Taylor R.W. (2003). Late-Onset Encephalopathy Associated with a C11777A Mutation of Mitochondrial DNA. Neurology.

[59] Scaglia F. (2010). The Role of Mitochondrial Dysfunction in Psychiatric Disease. Dev. Disabil. Res. Rev..

[60] Brown M.D., Shoffner J.M., Kim Y.L., Jun A.S., Graham B.H., Cabell M.F., Gurley D.S., Wallace D.C. (1996). Mitochondrial DNA Sequence Analysis of Four Alzheimer’s and Parkinson’s Disease Patients. Am. J. Med. Genet..

[61] Brandon M., Baldi P., Wallace D.C. (2006). Mitochondrial Mutations in Cancer. Oncogene.

[62] Ruiz-Pesini E., Lapena A.-C., Díez-Sánchez C., Pérez-Martos A., Montoya J., Alvarez E., Díaz M., Urriés A., Montoro L., López-Pérez M.J. (2000). Human MtDNA Haplogroups Associated with High or Reduced Spermatozoa Motility. Am. J. Hum. Genet..

[63] Kirby D.M., Kahler S.G., Freckmann M., Reddihough D., Thorburn D.R. (2000). Leigh Disease Caused by the Mitochondrial DNA G14459A Mutation in Unrelated Families. Ann. Neurol..

[64] Khurana D.S., Valencia I., Goldenthal M.J., Legido A. (2013). Mitochondrial Dysfunction in Epilepsy. Seminars in Pediatric Neurology.

[65] Fasterius E., Uhlén M., Al-Khalili Szigyarto C. (2019). Single-Cell RNA-Seq Variant Analysis for Exploration of Genetic Heterogeneity in Cancer. Sci. Rep..

[66] Blein S., Bardel C., Danjean V., McGuffog L., Healey S., Barrowdale D., Lee A., Dennis J., Kuchenbaecker K.B., Soucy P. (2015). An Original Phylogenetic Approach Identified Mitochondrial Haplogroup T1a1 as Inversely Associated with Breast Cancer Risk in BRCA2 Mutation Carriers. Breast Cancer Res..

[67] Melchionda L., Damseh N.S., Abu Libdeh B.Y., Nasca A., Elpeleg O., Zanolini A., Ghezzi D. (2014). A Novel Mutation in TTC19 Associated with Isolated Complex III Deficiency, Cerebellar Hypoplasia, and Bilateral Basal Ganglia Lesions. Front. Genet..

[68] Horváth A., Horáková E., Dunaj Íková P., Verner Z.K., Pravdová E.K., Lapetová I., Cuninková L., Lukeš J. (2005). Downregulation of the Nuclear-Encoded Subunits of the Complexes III and IV Disrupts Their Respective Complexes but Not Complex I in Procyclic Trypanosoma Brucei. Mol. Microbiol..

[69] Chagnon P., Gee M., Filion M., Robitaille Y., Belouchi M., Gauvreau D. (1999). Phylogenetic Analysis of the Mitochondrial Genome Indicates Significant Differences between Patients with Alzheimer Disease and Controls in a French-Canadian Founder Population. Am. J. Med. Genet..

[70] Boulet L., Karpati G., Shoubridge E.A. (1992). Distribution and Threshold Expression of the TRNA (Lys) Mutation in Skeletal Muscle of Patients with Myoclonic Epilepsy and Ragged-Red Fibers (MERRF). Am. J. Hum. Genet..

[71] Grossman L.I., Shoubridge E.A. (1996). Mitochondrial Genetics and Human Disease. Bioessays.

[72] Choi B.-O., Hwang J.H., Cho E.M., Jeong E.H., Hyun Y.S., Jeon H.J., Seong K.M., Cho N.S., Chung K.W. (2010). Mutational Analysis of Whole Mitochondrial DNA in Patients with MELAS and MERRF Diseases. Exp. Mol. Med..

[73] Graf W.D., Marin-Garcia J., Gao H.G., Pizzo S., Naviaux R.K., Markusic D., Barshop B.A., Courchesne E., Haas R.H. (2000). Autism Associated with the Mitochondrial DNA G8363A Transfer RNALys Mutation. J. Child Neurol..

[74] Wong L.J.C., Liang M.H., Kwon H., Bai R.K., Alper Ö., Gropman A. (2002). A Cystic Fibrosis Patient with Two Novel Mutations in Mitochondrial DNA: Mild Disease Led to Delayed Diagnosis of Both Disorders. Am. J. Med. Genet..

[75] Hung P., Wang H. (2007). A Previously Undescribed Leukodystrophy in Leigh Syndrome Associated with T9176C Mutation of the Mitochondrial ATPase 6 Gene. Dev. Med. Child Neurol..

[76] Blanco-Grau A., Bonaventura-Ibars I., Coll-Cantí J., Melià M.J., Martinez R., Martínez-Gallo M., Andreu A.L., Pinos T., García-Arumí E. (2013). Identification and Biochemical Characterization of the Novel Mutation m. 8839G> C in the Mitochondrial ATP6 Gene Associated with NARP Syndrome. Genes Brain Behav..

[77] Guo Y., Zhang Y., Li F., Liu P., Liu Y., Yang C., Song J., Zhang N., Chen Z. (2018). The Biochemical Characterization of a Missense Mutation m.8914C>T in ATP6 Gene Associated with Mitochondrial Encephalomyopathy. Int. J. Dev. Neurosci..

[78] Pronicka E., Piekutowska-Abramczuk D., Pronicki M. (2008). Mitochondrial Diseases in Children Including Leigh Syndrome--Biochemical and Molecular Background. Postepy Biochem..

[79] Houštěk J., Pícková A., Vojtíšková A., Mráček T., Pecina P., Ješina P. (2006). Mitochondrial Diseases and Genetic Defects of ATP Synthase. Biochim. Biophys. Acta (BBA)—Bioenerg..

[80] Ichikawa T., Arai M., Miyashita M., Arai M., Obata N., Nohara I., Oshima K., Niizato K., Okazaki Y., Doi N. (2012). Schizophrenia: Maternal Inheritance and Heteroplasmy of MtDNA Mutations. Mol. Genet. Metab..

[81] Krausz C., Escamilla A.R., Chianese C. (2015). Genetics of Male Infertility: From Research to Clinic. Reproduction.

[82] Wai T., Ao A., Zhang X., Cyr D., Dufort D., Shoubridge E.A. (2010). The Role of Mitochondrial DNA Copy Number in Mammalian Fertility. Biol. Reprod..

[83] Moscatelli N., Lunetti P., Braccia C., Armirotti A., Pisanello F., De Vittorio M., Zara V., Ferramosca A. (2019). Comparative Proteomic Analysis of Proteins Involved in Bioenergetics Pathways Associated with Human Sperm Motility. Int. J. Mol. Sci..

[84] Spiropoulos J., Turnbull D.M., Chinnery P.F. (2002). Can Mitochondrial DNA Mutations Cause Sperm Dysfunction?. Mol. Hum. Reprod..

[85] Ferramosca A., Focarelli R., Piomboni P., Coppola L., Zara V. (2008). Oxygen Uptake by Mitochondria in Demembranated Human Spermatozoa: A Reliable Tool for the Evaluation of Sperm Respiratory Efficiency. Int. J. Androl..

[86] Ferramosca A., Zara V. (2017). Mitochondria and Fertility: The Mitochondria Critical Role on Spermatozoa Function. JDREAM J. Interdiscip. Res. Appl. Med..

[87] Nakada K., Sato A., Yoshida K., Morita T., Tanaka H., Inoue S.I., Yonekawa H., Hayashi J.I. (2006). Mitochondria-Related Male Infertility. Proc. Natl. Acad. Sci. USA.

[88] Yano T., Magnitsky S., Ohnishi T. (2000). Characterization of the Complex I-Associated Ubisemiquinone Species: Toward the Understanding of Their Functional Roles in the Electron/Proton Transfer Reaction. Biochim. Biophys. Acta Bioenerg..

[89] Ambulkar P.S., Waghmare J.E., Chaudhari A.R., Wankhede V.R., Tarnekar A.M., Shende M.R., Pal A.K. (2016). Large Scale 7436-Bp Deletions in Human Sperm Mitochondrial DNA with Spermatozoa Dysfunction and Male Infertility. J. Clin. Diagn. Res..

[90] Ambulkar P.S., Chuadhari A.R., Pal A.K. (2016). Association of Large Scale 4977-Bp “Common” Deletions in Sperm Mitochondrial DNA with Asthenozoospermia and Oligoasthenoteratozoospermia. J. Hum. Reprod. Sci..

[91] Kao S.H., Chao H.T., Wei Y.H. (1998). Multiple Deletions of Mitochondrial DNA Are Associated with the Decline of Motility and Fertility of Human Spermatozoa. Mol. Hum. Reprod..

[92] Talebi E., Karimian M., Nikzad H. (2018). Association of Sperm Mitochondrial DNA Deletions with Male Infertility in an Iranian Population. Mitochondrial DNA A.

[93] Guo Z., Jin C., Yao Z., Wang Y., Xu B. (2017). Analysis of the Mitochondrial 4977 Bp Deletion in Patients with Hepatocellular Carcinoma. Balk. J. Med. Genet..

[94] Tanaka M., Ozawa T. (1992). Analysis of Mitochondrial DNA Mutations. Protocols in Molecular Neurobiology.

[95] Carra E., Sangiorgi D., Gattuccio F., Rinaldi A.M. (2004). Male Infertility and Mitochondrial DNA. Biochem. Biophys. Res. Commun..

[96] Chari M.G., Colagar A.H., Bidmeshkipour A. (2015). A Novel Large-Scale Deletion of The Mitochondrial of Spermatozoa of Men in North Iran. Int. J. Fertil. Steril..

[97] Barbagallo F., Vignera S.L., Cannarella R., Aversa A., Calogero A.E., Condorelli R.A. (2020). Evaluation of Sperm Mitochondrial Function: A Key Organelle for Sperm Motility. J. Clin. Med..

[98] St. John J.C., Jokhi R.P., Barratt C.L.R. (2001). Men with Oligoasthenoteratozoospermia Harbour Higher Numbers of Multiple Mitochondrial DNA Deletions in Their Spermatozoa, but Individual Deletions Are Not Indicative of Overall Aetiology. Mol. Hum. Reprod..

[99] Holyoake A.J., McHugh P., Wu M., O’Carroll S., Benny P., Sin I.L., Sin F.Y.T. (2001). High Incidence of Single Nucleotide Substitutions in the Mitochondrial Genome Is Associated with Poor Semen Parameters in Men. Int. J. Androl..

[100] Güney A.I., Javadova D., Kirac D., Ulucan K., Koc G., Ergec D., Tavukcu H., Tarcan T. (2012). Detection of Y Chromosome Microdeletions and Mitochondrial DNA Mutations in Male Infertility Patients. Genet. Mol. Res..

[101] Hosseinzadeh Colagar A., Karimi F. (2014). Large Scale Deletions of the Mitochondrial DNA in Astheno, Asthenoterato and Oligoasthenoterato-Spermic Men. Mitochondrial DNA.

[102] Ji J., Xu M., Huang Z., Li L., Zheng H., Yang S., Li S., Jin L., Ling X., Xia Y. (2017). Mitochondrial DNA Sequencing and Large-Scale Genotyping Identifies MT-ND4 Gene Mutation m.11696G>A Associated with Idiopathic Oligoasthenospermia. Oncotarget.

[103] Barbhuiya P.N., Gogoi A., Ahmed G., Mahanta R. (2016). Prevalence of Mitochondrial DNA Nucleotide Substitution Mutations in Male Infertile Cases of Northeast India. J. Infertil. Reprod. Biol..

[104] Khan A.U.H., Rathore M.G., Allende-Vega N., Vo D.N., Belkhala S., Orecchioni S., Talarico G., Bertolini F., Cartron G., Lecellier C.H. (2016). Human Leukemic Cells Performing Oxidative Phosphorylation (OXPHOS) Generate an Antioxidant Response Independently of Reactive Oxygen Species (ROS) Production. EBioMedicine.

[105] Zhang Y., Zhao Y., Wen S., Yan R., Yang Q., Chen H. (2017). Associations of Mitochondrial Haplogroups and Mitochondrial DNA Copy Numbers with End-Stage Renal Disease in a Han Population. Mitochondrial DNA A.

[106] Mughal I.A., Irfan A., Hameed A., Jahan S. (2016). Sperm Mitochondrial DNA 15bp Deletion of Cytochrome c Oxidase Subunit III Is Significantly Associated with Human Male Infertility in Pakistan. J. Pak. Med. Assoc..

[107] Al Smadi M.A., Hammadeh M.E., Solomayer E., Batiha O., Altalib M.M., Jahmani M.Y., Shboul M.A., Nusair B., Amor H. (2021). Impact of Mitochondrial Genetic Variants in ND1, ND2, ND5, and ND6 Genes on Sperm Motility and Intracytoplasmic Sperm Injection (ICSI) Outcomes. Reprod. Sci..

[108] Dahadhah F.W., Jaweesh M.S., Al Zoubi M.S., Alarjah M.I.A., Hammadeh M.E., Amor H. (2021). Lack of Association between Single Polymorphic Variants of the Mitochondrial Nicotinamide Adenine Dinucleotide Dehydrogenase 3, and 4L (MT-ND3 and MT-ND4L) and Male Infertility. Andrologia.

[109] Dahadhah F.W., Saleh Jaweesh M., Salim Al Zoubi M., Issam Abu Alarjah M., Eid Hammadeh M., Amor H. (2021). Mitochondrial Nicotinamide Adenine Dinucleotide Hydride Dehydrogenase (NADH) Subunit 4 (MTND4) Polymorphisms and Their Association with Male Infertility. J. Assist. Reprod. Genet..

[110] Saleh Jaweesh M., Eid Hammadeh M., Dahadhah F.W., Salim Al Zoubi M., Amor H. (2022). Association between the Single Nucleotide Variants of the Mitochondrial Cytochrome B Gene (MT-CYB) and the Male Infertility. Mol. Biol. Rep..

[111] Kao S.-H., Chao H.-T., Wei Y.-H. (1995). Mitochondrial Deoxyribonucleic Acid 4977-Bp Deletion Is Associated with Diminished Fertility and Motility of Human Sperm. Biol. Reprod..

[112] Ieremiadou F., Rodakis G.C. (2009). Correlation of the 4977 Bp Mitochondrial DNA Deletion with Human Sperm Dysfunction. BMC Res. Notes.

[113] Al Zoubi M.S., Al-Batayneh K., Alsmadi M., Rashed M., Al-Trad B., Al Khateeb W., Aljabali A., Otoum O., Al-Talib M., Batiha O. (2020). 4,977-Bp Human Mitochondrial DNA Deletion Is Associated with Asthenozoospermic Infertility in Jordan. Andrologia.

[114] Monnot S., Samuels D.C., Hesters L., Frydman N., Gigarel N., Burlet P., Kerbrat V., Lamazou F., Frydman R., Benachi A. (2013). Mutation Dependance of the Mitochondrial DNA Copy Number in the First Stages of Human Embryogenesis. Hum. Mol. Genet..

[115] Vanniarajan A., Govindaraj P., Carlus S.J., Aruna M., Aruna P., Kumar A., Jayakar R.I., Lionel A.C., Gupta S., Rao L. (2011). Mitochondrial DNA Variations Associated with Recurrent Pregnancy Loss among Indian Women. Mitochondrion.

[116] Diez-Juan A., Rubio C., Marin C., Martinez S., Al-Asmar N., Riboldi M., Díaz-Gimeno P., Valbuena D., Simón C. (2015). Mitochondrial DNA Content as a Viability Score in Human Euploid Embryos: Less Is Better. Fertil. Steril..

[117] Rani D.S., Vanniarajan A., Gupta N.J., Chakravarty B., Singh L., Thangaraj K. (2006). A Novel Missense Mutation C11994T in the Mitochondrial ND4 Gene as a Cause of Low Sperm Motility in the Indian Subcontinent. Fertil. Steril..

[118] Mao G.H., Huang X.H., Geng X.J., Li Q., Zhang Y., Dou Q. (2020). Correlation between Sperm Mitochondrial ND5 and ND6 Gene Variations and Total Fertilisation Failure. Arch. Med. Sci..

[119] Lestienne P., Reynier P., Chrétien M.F., Penisson-Besnier I., Malthièry Y., Rohmer V. (1997). Oligoasthenospermia Associated with Multiple Mitochondrial DNA Rearrangements. Mol. Hum. Reprod..

[120] Vertika S., Singh K.K., Rajender S. (2020). Mitochondria, Spermatogenesis, and Male Infertility—An Update. Mitochondrion.

[121] Zhang J.L., Mao G.H., Huang X.H., Chang H.Y., Zheng Y., Cao X. (2018). Association between Sperm Mitochondrial ND2 Gene Variants and Total Fertilization Failure. Syst. Biol. Reprod. Med..

[122] Wu H., Whitcomb B.W., Huffman A., Brandon N., Labrie S., Tougias E., Lynch K., Rahil T., Sites C.K., Richard Pilsner J. (2019). Associations of Sperm Mitochondrial DNA Copy Number and Deletion Rate with Fertilization and Embryo Development in a Clinical Setting. Hum. Reprod..

[123] Pereira L., Gonçalves J., Bandelt H.J. (2008). Mutation C11994T in the Mitochondrial ND4 Gene Is Not a Cause of Low Sperm Motility in Portugal. Fertil. Steril..

